# *Bacillus thuringiensis* and its pest control potential as endophyte

**DOI:** 10.1002/ps.70771

**Published:** 2026-03-28

**Authors:** Maria Giovanna De Luca, Giovanni Jesu, Daniele Bruno, Elia Russo, Andrea Becchimanzi, Gianluca Tettamanti, Maria Cristina Digilio, Ilaria Di Lelio, Francesco Pennacchio

**Affiliations:** ^1^ Department of Agricultural Sciences University of Naples Federico II Portici (Na) Italy; ^2^ Department of Biotechnology and Life Sciences University of Insubria Varese Italy; ^3^ Interuniversity Center for Studies on Bioinspired Agro‐Environmental Technology (BAT Center), University of Naples Federico II Portici (Na) Italy

**Keywords:** bioinsecticides, biological control, endophytes, insect–plant–microbe interactions

## Abstract

**BACKGROUND:**

*Bacillus thuringiensis* (*Bt*) is widely employed as bioinsecticide due to its selectivity and efficacy against pest insects. While its entomopathogenic activity has been extensively documented, its ecology and potential role as a plant endophyte have been much less explored. Here we investigate if *Bt,* isolated from a registered commercial bioinsecticide, can develop as endophyte in tomato plants and the resulting effects on *Spodoptera littoralis* larvae.

**RESULTS:**

Tomato plants inoculated with *Bt,* obtained from a commercial formulation, were systemically colonized by seedling drenching, with spores ensuring the highest efficiency of colonization. Feeding assays on *Bt*‐colonized plants showed a marked reduction of survival in early instars of *S. littoralis* larvae, reproducing midgut alterations induced by *Bt* toxins, such as epithelial disruption, mitochondrial disorganization, and alterations of the peritrophic matrix. Surviving larvae completed the development but displayed reduced immunocompetence, decreased adult longevity, and lower fertility. The reduced immunocompetence increased larval susceptibility to foliar spray applications of *Bt‐*based bioinsecticides, even in later instars poorly sensitive, revealing a synergistic interaction between plant endophytic colonization and spray treatments.

**CONCLUSION:**

This study demonstrates that endophytic colonization of tomato plants by *Bt* negatively affects *S. littoralis* fitness, making its larval stages also more sensitive to spray applications of *Bt* and, likely, to the action of other biocontrol agents. These findings open new perspectives for *Bt* use in sustainable pest management strategies. © 2026 The Author(s). *Pest Management Science* published by John Wiley & Sons Ltd on behalf of Society of Chemical Industry.

## Graphical abstract

*Bacillus thuringiensis* (Bt) systemically colonizes tomato plants as endophyte, inducing midgut damage, fitness reduction and immune suppression in *Spodoptera littoralis* larvae feeding on *Bt*‐colonized leaves. The immune suppression enhances the susceptibility to *Bt* treatments, allowing a synergistic dual use of *Bt* commercial formulations, by combined seedling drenching and foliar spray.

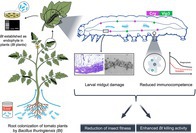

## 1 INTRODUCTION

*Bacillus thuringiensis* (*Bt*) is a Gram‐positive, aerobic, spore‐forming, entomopathogenic bacterium, ubiquitous in soil environments and widely employed as a bioinscticide in integrated pest management (IPM) programmes.^1^ Its insecticidal activity is highly selective, targeting specific insect orders (Lepidoptera, Coleoptera, and Diptera), without undesired negative effects on beneficial insects and, more in general, on non‐target species.^2^, ^3^ In Lepidoptera, the δ‐endotoxins in the Cry family and the vegetative insecticidal proteins Vip3 are particularly effective against several pest species.^2^, ^3^ Cry toxins in the parasporal crystals, produced during sporulation, are solubilized and proteolytically activated in the larval midgut, where they bind to gut epithelial receptors, inducing pore formation, osmotic shock and cell lysis, followed by septicemia, and ultimately death.^4^, ^5^, ^6^ Vip3 proteins trigger a similar midgut damage but bind to distinct receptors, making them active against Cry‐resistant strains.^7^ The in‐depth study on *Bt* toxins has allowed the development of widely used bioinsecticides and insect‐resistant transgenic plants, which successfully dominate the global market.^8^, ^9^, ^10^

Despite the widespread *Bt* use as an entomopathogen, its ecology has been comparatively much less investigated. *Bt* is ubiquitous in the soil, but it seems that the soil is not a mere reservoir of spores dispersed in the environment to infect new hosts.^11^ Some studies show that *Bt* can inhabit plant tissues as endophyte. Both naturally occurring endophytic strains,^12^, ^13^ and those artificially established *in planta via* rhizospheric inoculation^14^, ^15^, ^16^ can exert direct insecticidal activity, while *Bt*‐colonized plants can activate endogenous defence barriers against insects, pathogens, and nematodes.^12^, ^17^, ^18^, ^19^, ^20^, ^21^ Remarkably, this induced resistance can also protect against sap‐sucking insects,^22^ which are generally non‐susceptible to *Bt* toxins, with rare exceptions.^23^ However, the low level of information on endophytic colonization and on mechanisms underlying the resulting insecticide activity of colonized plants currently limit the possible development of novel strategies of plant protection based on a more stable and systemic presence of *Bt* within plant tissues,^24^ which could be a possible alternative to transgenic plants, especially in those countries where their use is not allowed.

To address this gap and explore this opportunity, we investigated if and how a commercial formulation containing *Bt* subsp. *aizawai* can establish endophytic colonization in tomato plants, which, when colonized, were used to study the physiological and developmental alterations induced on lepidopteran larvae (*Spodoptera littoralis*) feeding on them, thereby providing a tractable framework to begin shedding light on the still poorly understood basis of endophytic *Bt*‐mediated effects on insects. Based on the information developed, we propose a new use of a commercial *Bt* formulation, which, by combining endophytic colonization and conventional spray delivery, enhances insect control activity, and reduces the risk of resistance insurgence. The use of a registered *Bt* commercial product will allow us to implement more rapidly in the field any plant protection strategy based on its use as an endophyte.

## 2 MATERIALS AND METHODS

### 2.1 Plant material and endophytic colonization

*Solanum lycopersicum* cv ‘Dwarf San Marzano’ plants colonized by *Bacillus thuringiensis* (*Bt*‐plants) and control plants (C‐plants) were obtained as hereafter described. *Bacillus thuringiensis* subsp. *Aizawa*i, strain ABTS‐1857, was isolated from the commercial formulation XenTari® (XTR – Valent BioSciences Corporation, Libertyville, Illinois, USA) and used to obtain distinct inoculum preparations as described in Supporting Methods S1. Tomato seedlings were drenched with 0.1% Tween 20 solutions containing either 1 mg/mL of XenTari® (XTR ‐ Valent BioSciences Corporation, Libertyville, Illinois, USA), or suspensions, prepared from XTR, containing vegetative cells (1 × 10^7^ cells/mL), spores (1 × 10^7^ spores/mL), or a mixture (Mix) composed of equal parts of vegetative cells and spores (total concentration 1 × 10^7^ units/mL). In all cases, the microbial concentration corresponded to that contained in 1 mg of XTR. Control plants received only Tween 20 solution (C). Further details on experimental procedures are reported in Supporting Methods 2.

Plants were maintained at 25 ± 1 °C and 70 ± 5% RH, under a 16:8 h light/dark cycle. To assess endophytic colonization, 4‐week‐old experimental plants were collected, carefully washed under running tap water, primarily performed on roots to remove adhering coarse soil particles, surface‐sterilized by immersion in a solution of 1% NaOCl for 5 min, in 70% EtOH for 1 min, and, then, rinsed three times with sterile ddH₂O. Sterilized plant tissues were dissected, plated on LB agar, and incubated at 30 °C in the dark for 48 h. Sterilization efficacy was checked by plating 100 μL of each final rinse water on LB agar plates. Samples were collected from five separate experimental plants. Colonization was expressed as a percentage of colonized plants and as colonization frequency, calculated as the percentage of colonized tissue sections per plant (Supporting Methods 3).^25^ Emerging bacterial colonies were sub‐cultured on fresh LB agar for morphological characterization^26^ and molecular identification through PCR amplification of the *Cry1* gene transcript^27^ (Supporting Methods 4 and Supporting Table S1); the *Cry1* amplicon generated from *Bt* colonies isolated from XenTari® (Supporting Methods S1) was used as a positive control.

### 2.2 Insect bioassays

Bioassays were performed on newly hatched *S. littoralis* larvae fed with sub‐apical leaves of 4‐week‐old *Bt*‐plants (*Bt*‐larvae) or of C‐plants (C‐larvae) to assess their impact on insect survival and development. Leaf discs were offered to larvae in plastic multi‐well rearing trays (RT32W, Frontier Agricultural Sciences, Pitman, NJ, USA) as previously described,^28^ maintained in an environmental chamber at 25 ± 1 °C, 70 ± 5% RH, and 16:8 h light/dark photoperiod (Supporting Methods 5).

Larval survival and development were daily monitored in bioassays started with newly hatched larvae, using six experimental groups of 20 larvae per treatment. This bioassay was replicated six times.

On surviving 3^rd^ instar larvae, the following parameters were recorded: larval survival and weight, leaf consumption (on the first day of 4^th^, 5^th^ and 6^th^ instars), cumulative pupal survival, time to adult emergence, cumulative adult survival and total fertility. Leaf consumption was calculated as the difference between the initial and final weight of the leaf disks. The loss of water assessed was very limited and equal in all treatments and then, not considered. These data were collected from two experimental replicates, using experimental groups of 16 larvae each. To confirm the effective *Bt* presence in the gut of experimental larvae, RT‐PCR was used to detect *Cry1* and *Vip3* transcripts as detailed in Supporting Methods 6.

### 2.3 Light and transmission electron microscopy

The 2^nd^ instar experimental larvae, which showed the highest mortality rate in the feeding bioassay, were anesthetized on ice and then dissected to isolate the midgut with the enclosed intestinal content. To evaluate any structural and ultrastructural change in the midgut induced by the ingestion of *Bt‐*plant leaves, midgut samples derived *Bt*‐larvae were analyzed by light and transmission electron microscopy (TEM) and compared with those derived from C‐larvae. Sample preparation protocols are reported in Supporting Methods 7.

### 2.4 Assessment of immune competence

To investigate whether the exposure to *Bt* of experimental larvae of *S. littoralis* induced a reduced immunocompetence, as previously reported,^29^ despite the absence of increased mortality, cellular immune competence was evaluated using established protocols, slightly modified, to measure encapsulation, nodulation, and phagocytosis responses in surviving 6^th^ instars^30^, ^31^ Detailed procedures are provided in Supporting Methods 8.

### 2.5 Assessing lethality of *Bt*‐based commercial product on *Bt*‐larvae

The effect of sublethal doses (sld) of the commercial *Bt*‐based formulation XTR (XTR‐sld) on experimental *Bt*‐larvae were evaluated both on 2nd instar larvae, which exhibited high mortality rates in feeding bioassays, and on 4^th^ instar larvae, which survived after feeding on *Bt*‐colonized leaves. For each bioassay, 16 larvae were individually transferred to multi‐well plastic rearing trays prepared as described above and fed *ad libitum* for 3 days with experimental tomato leaves, uniformly sprayed either with XTR or water (control). Preliminary bioassays were performed to determine the sublethal XTR doses (i.e., causing only growth retardation and/or weight decrease but no mortality) in C‐larvae. The resulting sublethal doses were 0.1 μg cm^−2^ for 2^nd^ instar and 1 μg cm^−2^ for 4^th^ instar. During the bioassay, the larvae were fed on leaves treated with these sublethal doses, and mortality was recorded daily for 8 consecutive days. All bioassays were performed twice.

### 2.6 Statistical analysis

Colonization of plants was analyzed using Fisher's exact test (Agresti–Coull correction), while differences among tissue colonization were evaluated with one‐way ANOVA or, when assumptions were not met, with the Kruskal–Wallis test. Adult longevity, fertility, circulating viable hemocytes, encapsulation, phagocytosis, and nodulation were compared using unpaired Student's t‐tests or Mann–Whitney tests, as appropriate. Survival curves of *S. littoralis* larvae, pupae, and XTR bioassays were analyzed with Kaplan–Meier and Log‐rank tests. Data normality and homoscedasticity were verified with D'Agostino–Pearson and Bartlett's tests, respectively. All analyses were carried out with GraphPad Prism v6.0b (GraphPad Software Inc.).

## 3 RESULTS

### 3.1 *Bt* endophytic colonization and its impact on *S. littoralis*

**Figure 1:**
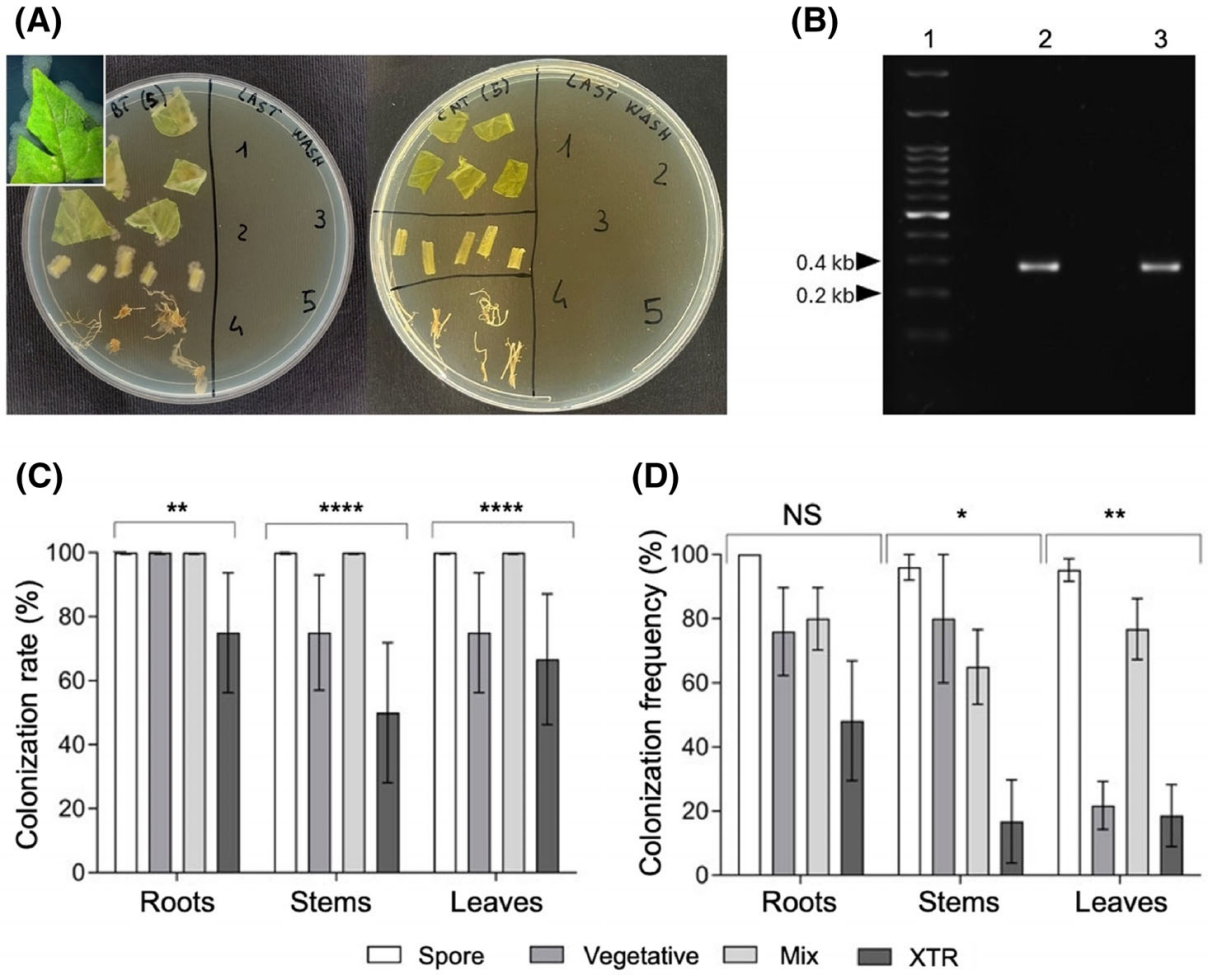
Endophytic colonization of tomato plants by *Bacillus thuringiensis*. (A) *In vitro* procedure used to verify plant endophytic colonization and the effectiveness of surface sterilization. Left Petri dish: surface‐sterilized tissue sections (leaves, stems, and roots) from five tomato plants were plated on LB agar to detect the development of *Bt* colonies from internal tissues; the inset shows the typical appearance of a *Bt* colony developed from leaf tissue. Right Petri dish (controls): surface‐sterilized tissue sections from five C‐plants were plated in parallel. The final rinse (10 μL aliquots) from each plant (numbered 1–5) were spotted onto LB agar to confirm the absence of surface contamination. (B) Agarose gel electrophoresis (1% w/v) of *Cry1* gene amplification products obtained from bacterial colonies re‐isolated from *Bt*‐colonized tomato leaves. Individual colonies were picked from plated leaf samples and resuspended in sterile distilled water before PCR amplification; Lane 1: molecular marker; Lanes 2 and 3: *Cry* gene amplification products from plant tissues and XTR, respectively. (C) Endophytic colonization rate (% of colonized plants) detected across different organs of plants treated with inocula containing spores, vegetative cells, or a mix of both, isolated from Xentari XTR (Fisher's exact test between treatment: Roots: *P* = 0.008; Stems: *P* < 0.0001; Leaves: *P* < 0.0001). (D) colonization frequency (% of colonized tissue sections per plant) expressed as means ± SE (one‐way ANOVA: roots, F _(4,20)_ = 6.809, *P* = 0.0782; stems, F _(4,20)_ = 10.56, *P* = 0.0144; leaves, F _(4,20)_ = 8.85, *P* = 0.0020). In all graphs asterisks denote statistical significance (*: *P* < 0.05; **: *P* < 0.01; ****: *P* < 0.0001).

The endophytic colonization of tomato plants by *Bt* was assessed by its re‐isolation from surface‐sterilized leaves, stems, and roots of inoculated plants (*Bt*‐plants) and from controls (C‐plants). Sterilized plant tissues were plated on LB agar, where characteristic *Bt* whitish, cream‐colored colonies with irregular edges^26^ were consistently observed around the plated samples obtained from *Bt*‐plants, while no colonies developed from C‐plant tissues (Fig. 1(A)). This was further corroborated by unequivocal molecular evidence obtained by PCR amplification of the *Cry1* gene from plant tissues (Fig. 1(B)). Spore and Mix inocula proved to be the most effective, resulting in a complete (100%) colonization of roots, stems, and leaves. Vegetative cells also ensured 100% root colonization rate, which dropped to 80% in stems and leaves; XTR performed less consistently, but still with 80% colonization in roots, 40% in stems, and 60% in leaves. The differences between treatments were highly significant across all plant tissues (Fisher's exact test: Roots, *P* = 0.008, Stems and Leaves, *P* < 0.0001; Fig. 1(C)). A similar trend was observed for colonization frequency (one‐way ANOVA: roots, *P* = 0.0782; stems, *P* = 0.0144; leaves, *P* = 0.0020, Fig. 1(D)), with spores exhibiting a significantly higher colonization frequency in all tissues. The lower and more variable performance of XTR is presumably attributable to its commercial formulation, optimized for spore–crystal stability and surface persistence, while vegetative cells and spores were freshly produced in culture and immediately used, a condition which may favor endophytic colonization of plants. Overall, these findings demonstrate that tomato plants can be efficiently and rapidly colonized also by *Bt* isolated from a commercial formulation, with spore‐based inocula representing the most effective tool for achieving systemic endophytic establishment.

**Figure 2:**
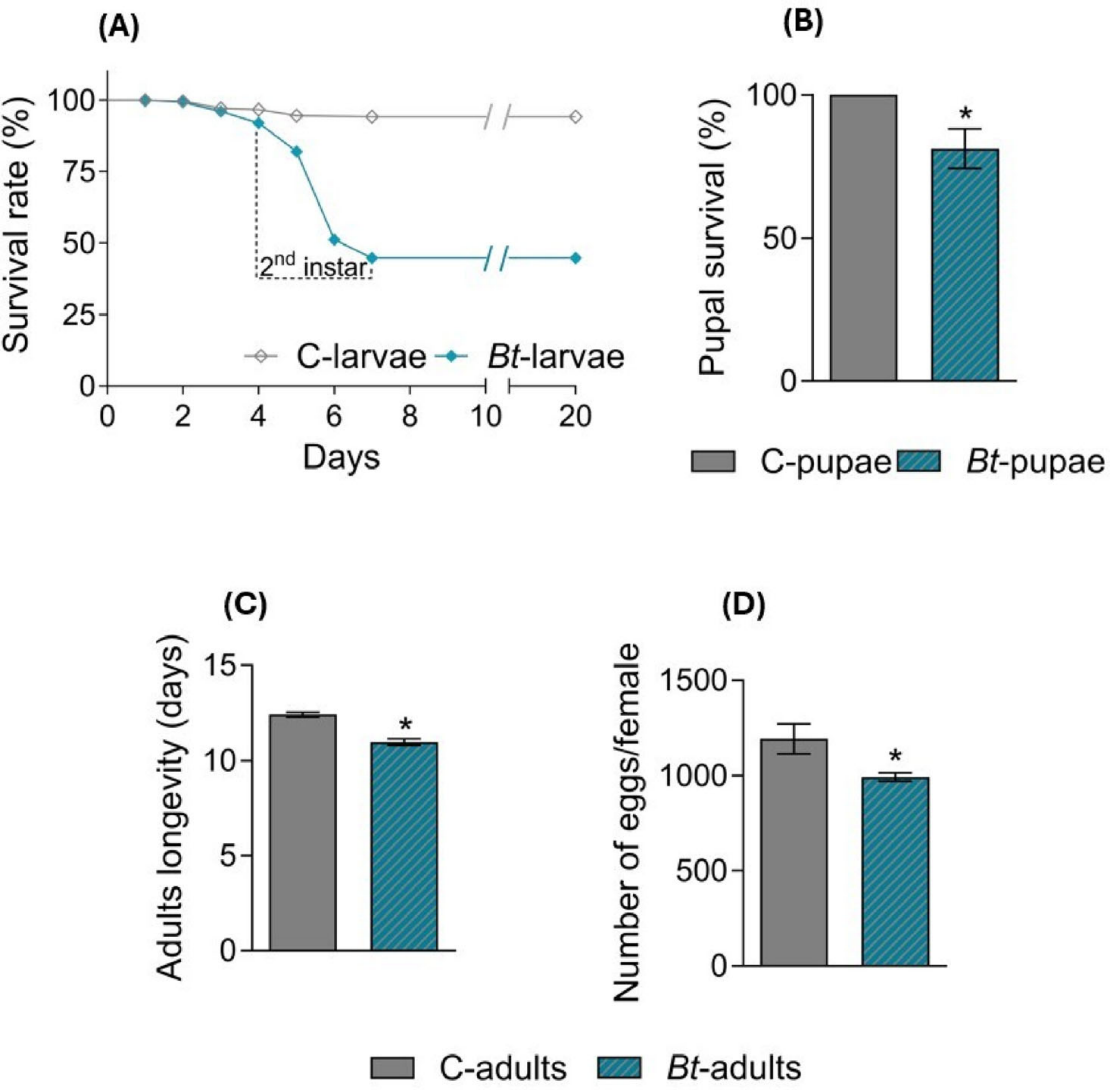
*Spodoptera littoralis* survival, longevity and fertility on *Bt*‐plants. (A) Survival rate of *Bt‐*larvae and C‐larvae (Log‐rank test: χ^2^ = 123.7, dF = 1, *P* < 0.0001, *n =* 240). (B) Cumulative pupal survival (Log‐rank test: χ^2^ = 6.517, dF = 1, *P* < 0.01, *n =* 32). (C) Adult longevity (Student's t‐test: t = 6.953, dF = 56, *P* < 0.0001, N_C_ = 32, N_*Bt*_ = 25). (D) Cumulative number of eggs laid per female (Mann–Whitney test: *P* < 0.01). The values are expressed as means ± SE. The asterisk denotes a statistically significant difference (**P < 0.01; ****P < 0.0001).

Larval survival was strongly affected by feeding on *Bt*‐colonized leaves, with only 45% of experimental larvae reaching the 3^rd^ instar (Fig. 2(A); Log‐rank test: *P* < 0.0001). Surviving larvae regularly developed and attained the pupal stage but showed reduced pupal survival (Fig. 2(B); Log‐rank test: *P* < 0.01), together with decreased adult longevity (Fig. 2(C); Student's *t*‐test: *P* < 0.0001) and cumulative fertility (Fig. 2(F); Mann–Whitney test: *P* < 0.01).

### 3.2 Effects of *Bt‐*plants on midgut morphology

**Figure 3:**
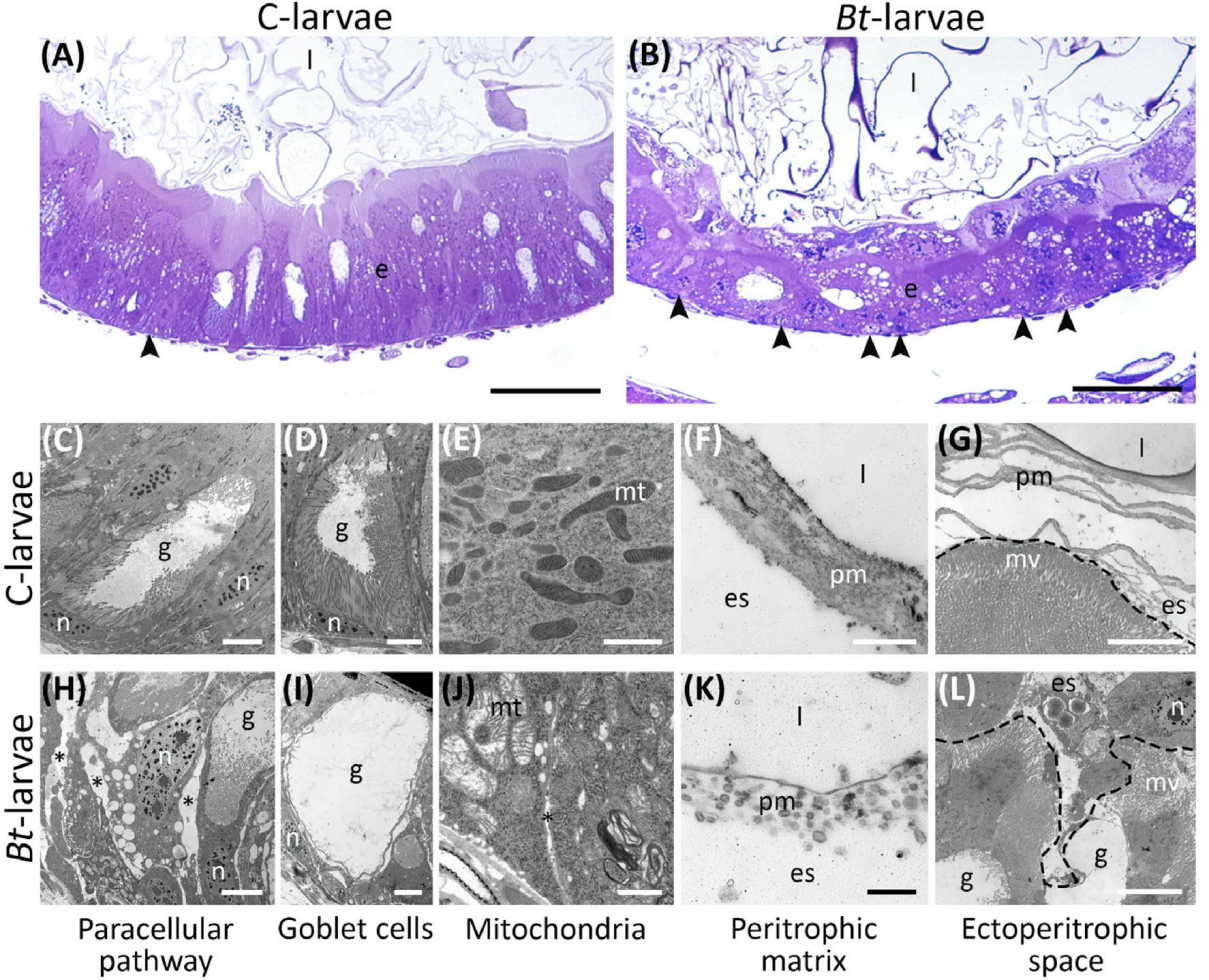
Effects on midgut morphology of *Spodoptera littoralis* larvae fed on *Bt‐*plant leaf disks. (A, B) Cross‐sections of the midgut epithelium; (C–L) TEM analysis. Arrowheads: stem cells; asterisks: gaps among adjacent cells; dashed line: apical region of the epithelium; e: epithelium; es: ectoperitrophic space; g: goblet cell; l: lumen; n: nucleus; mt: mitochondria; mv: microvilli; pm: peritrophic matrix. Bars: 20 μm (A, B), 10 μm (L), 5 μm (C, D, G–I), 2 μm (E, F), 1 μm (J, K).

Midgut morphology markedly differed between controls and *Bt*‐larvae (Fig. 3). In contrast to the intact midgut epithelium observed in C‐larvae (Fig. 3(A), (C)–(G)), *Bt*‐larvae displayed the characteristic lesions caused by Cry and Vip3 toxins,^6^, ^7^, ^32^, ^33^ including epithelial disruption with gaps in the paracellular pathway (Fig. 3(H), (J)), abnormal goblet cells with partial or complete loss of microvilli (Fig. 3(H), (I)), and marked proliferation of stem cells (Fig. 3(B)). In addition, TEM analyses revealed severe mitochondrial disorganization (Fig. 3(J)) and a fragmented, loosely compacted peritrophic matrix with ectoperitrophic leakage (Fig. 3(K)–(L)).

### 3.3 Impact of endophytic colonization on cellular immune response by *S. littoralis* larvae

**Figure 4:**
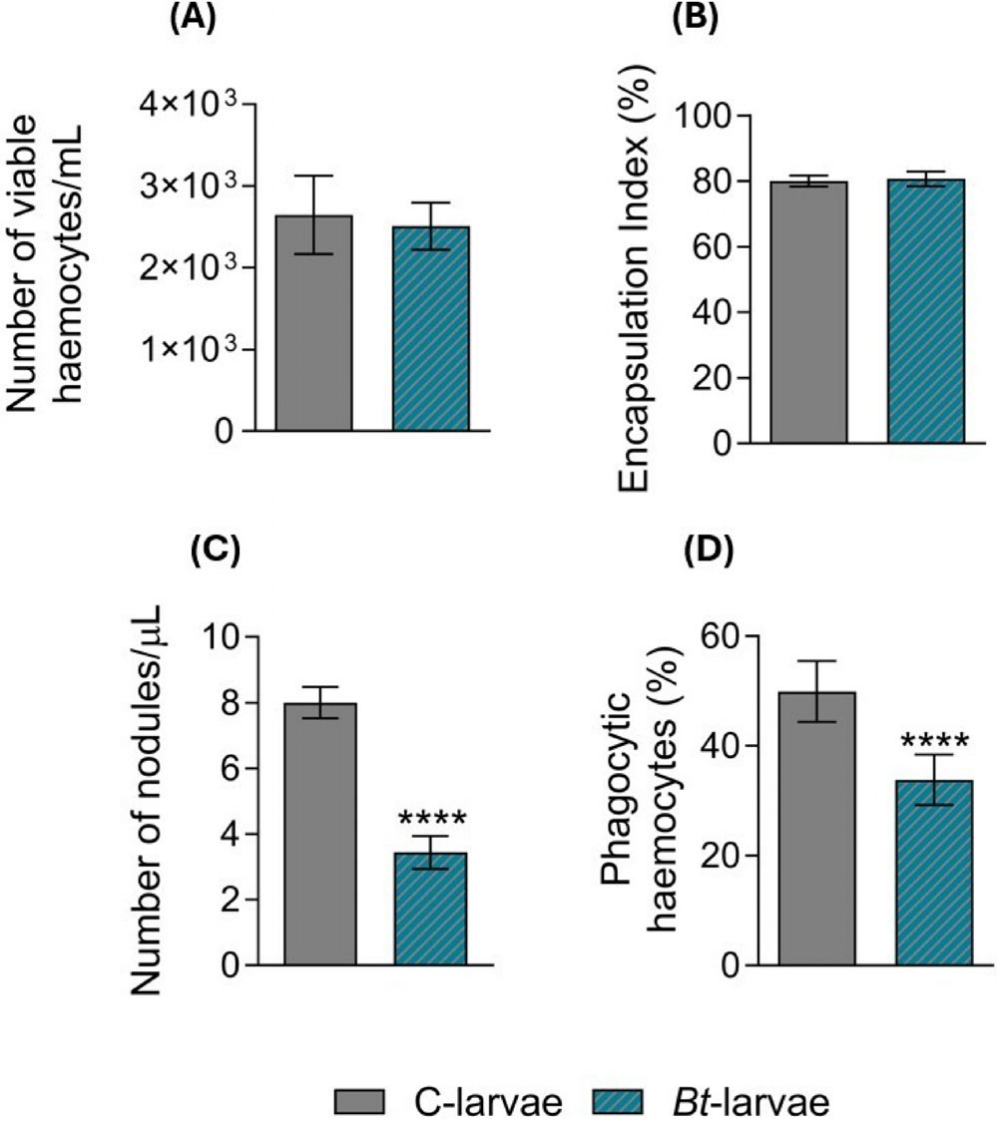
Cellular immunity in *Spodoptera littoralis* mature larvae as affected by feeding on *Bt* plants. (A) The number of circulating viable hemocytes remained unaltered in *Bt*‐larvae compared to C‐larvae (Student's t‐test: t = 1.335; dF = 58; *P* = 0.1872; *n =* 30). (B) *In vivo* encapsulation index of chromatographic beads did not differ between *Bt‐*larvae and C‐larvae (Student's t‐test: t = 1.413, dF = 66, *P* = 0.1624, *n =* 34). (C) The total number of nodules formed after injection of bacteria *in vivo* was significantly lower in *Bt*‐larvae compared to controls (Student's t‐test: t = 6.563, dF = 39, *P* < 0.0001, N_C_ = 22, N_*Bt*_ = 19). (D) The percentage of phagocytic hemocytes *in vitro* was significantly reduced in *Bt*‐larvae (Student's t‐test: t = 21.25, dF = 178, *P* < 0.0001, *n =* 30). The asterisk denotes statistically significant differences (****P < 0.0001).

RT‐PCR analysis confirmed that surviving 6^th^ instar larvae feeding on *Bt*‐plants harbored active *Bt* in their midgut (Supporting Fig. S1). This is suggestive of the possible occurrence of limited gut lesions and microbiome changes, associated with systemic immune dysregulation in *S. littoralis* larvae.^5^ To test this hypothesis, we carried out hemocyte counts (≈ 2.7 × 10^3 cells/μL per larva, > 98% viability across groups) and assessed their functionality through encapsulation, nodulation, and phagocytosis assays. *Bt*‐larvae exhibited a clear reduction in overall immunocompetence. However, while hemocyte number did not change in response to feeding on *Bt*‐plants (Fig. 4(A)), and the encapsulation response remained unaffected (Fig. 4(B); Student's t*‐test: P =* 0.1624), hemocytes displayed a marked reduction in nodulation capacity (Fig. 4(C); *P* < 0.0001) and showed impaired phagocytic activity of fluorescent bacteria (Fig. 4(D); *P* < 0.0001).

### 3.4 Enhancing the killing activity of *Bt*‐based commercial insecticides

**Figure 5:**
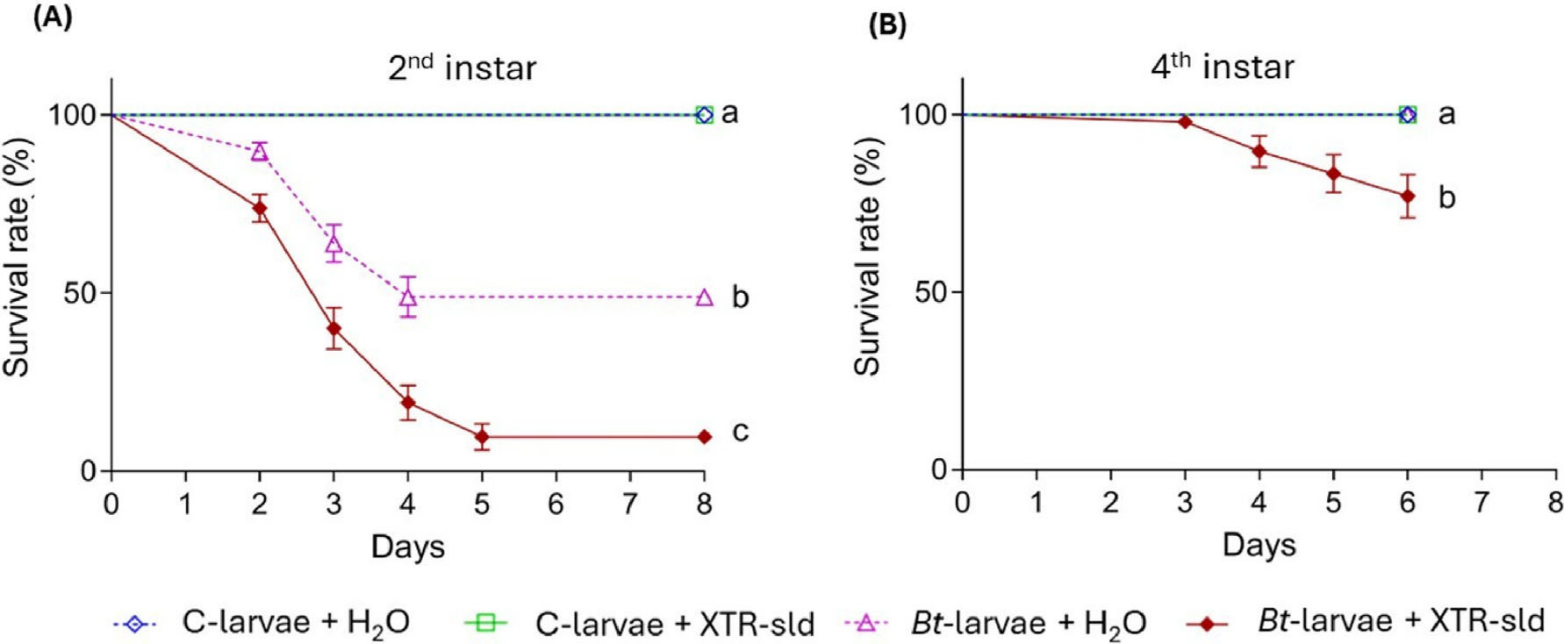
Susceptibility of *Bt‐*larvae to sublethal doses of XTR (XTR‐sld). **(A)** Survival of 2^nd^ instar larvae was drastically reduced in *Bt*‐larvae + XTR‐sld group compared with all other treatments (Log‐rank test: χ^2^ = 209.2, dF = 3, *P* < 0.0001, *n =* 200). Control larvae (C‐larvae + H₂O and C‐larvae + XTR‐sld) showed a survival rate 100%, significantly higher than both *Bt*‐larvae + H₂O (χ^2^ = 52.89, dF = 1, *P* < 0.0001) and *Bt*‐larvae + XTR‐sld (χ^2^ = 126.8, dF = 1, *P* < 0.0001); *Bt*‐larvae + XTR‐sld had lower survival than *Bt*‐larvae + H₂O (χ^2^ = 28.83, dF = 1, *P* < 0.0001). **(B)** In 4^th^ instar larvae, *Bt*‐larvae + XTR also showed significantly reduced survival compared with all other treatments (Log‐rank test: χ^2^ = 36.42, dF = 1, *P* < 0.0001, *n =* 56).

Given the reduced immunocompetence of *Bt*‐larvae, and the critical role of septicemia in *Bt*‐induced mortality,^5^ we investigated whether they were more sensitive to oral ingestion of *Bt* toxin. The susceptibility of *Bt*‐ and C‐larvae to a sublethal dose (sld) of the commercial bioinsecticide XTR (XTR‐sld) was evaluated. The 2^nd^ instar *Bt*‐larvae fed on tomato leaves treated with 0.1 μg cm^−2^ XTR (*Bt*‐larvae + XTR‐sld) showed a significant reduction in survival compared with all other treatments (Log‐rank test: *P* < 0.0001), reaching nearly 100% mortality within 5 days (Fig. 5(A)). Survival of larvae fed on control leaves treated with water (C + H₂O) or XTR‐sld (C‐larvae + XTR‐sld) remained unaffected (100% survival), and was significantly higher than in larvae fed on *Bt*‐plant leaves treated with either water (*Bt*‐larvae + H₂O) or with XTR‐sld (*Bt*‐larvae + XTR‐sld) (Fig. 5(A); Log‐rank test: *P* < 0.0001). *Bt‐larvae* + XTR‐sld exhibited significantly lower survival than *Bt‐larvae* + H₂O (Fig. 5(A); Log‐rank test: *P* < 0.0001), since feeding on *Bt* leaves negatively affected survival of early larval instars (Fig. 2(A)). Similarly, 4^th^ instar larvae showed significantly reduced survival when fed on *Bt*‐plant leaves treated with XTR‐sld (Fig. 5(B); Log‐rank test: *P* < 0.0001).

## 4 DISCUSSION AND CONCLUSIONS

In this study, we demonstrated that *Bt* isolated from a commercially available formulation is able to endophytically colonize tomato plants and to exert biologically relevant effects on the lepidopteran pest *S. littoralis*. The systemic endophytic establishment was confirmed by re‐isolation from surface‐sterilized tissues and molecular detection of *Cry1* gene, approaches that are widely used to assess bacterial endophytism.

Although we did not directly visualize *Bt* within plant tissues in this study, endophytic colonization has already been extensively documented in previous studies using labelled strains and microscopy, which demonstrated internal localization, systemic distribution, and persistence of the bacterium in a broad range of host plants, including both herbaceous crops and woody species, as well as the presence of metabolically active vegetative cells within plant tissues.^14^, ^16^, ^22^, ^24^, ^34^, ^35^, ^36^, ^37^, ^38^ Notably, several studies have also reported mortality in insects feeding on Bt‐colonized plants, whereas the functional mode of action within the insect has remained comparatively less well resolved.^12^, ^22^, ^34^, ^35^, ^36^, ^37^, ^38^

Building on this established body of evidence, the novelty of our work lies in addressing a key knowledge gap by disentangling the direct mode of action of endophytic *Bt* on larvae, linking plant colonization to functional outcomes in insect physiology and, ultimately, susceptibility to *Bt*‐based control measures.

Among the inocula we tested, spores proved to be most effective propagules for achieving widespread colonization of tomato plants, with a marked prevalence in roots compared to aerial tissues. Such patterns may reflect the higher nutrient availability, which creates a favorable microenvironment in the rhizosphere, promoting *Bt* colonization and persistence.^39^

Feeding bioassays revealed a strong negative impact of *Bt‐* plant on early larval instars of *S. littoralis*, which exhibited high mortality during the first developmental stages. This result aligns with the well‐documented susceptibility of younger larvae to *Bt* toxins^2^, ^3^, ^40^ and supports the hypothesis that ingestion of *Bt*‐colonized plant tissues exposes *S. littoralis* larvae to biologically active bacterial toxins. Although surviving larvae completed their development, they displayed altered life‐history traits, including reduced adult longevity and fertility. Morphological analysis revealed structural and ultrastructural alterations in the midgut of *Bt‐*larvae, including significant epithelial disruption, loss of cell integrity, and alterations in the peritrophic matrix, which is crucial for maintaining gut physiology.^41^ This pattern of injury is similar to that observed in larvae after ingestion of *Bt* toxins, including gaps in the paracellular pathway and loss of microvilli integrity,^6^, ^7^, ^32^, ^33^ resulting in a leaky gut that compromises nutrient absorption and allows resident bacteria to invade the haemocoel, initiating septicaemia.^42^, ^43^

However, the level of bacterial toxins in *Bt*‐plants is probably not sufficiently high to cause complete mortality. The development of surviving larvae, indeed, is likely due to midgut regeneration through stem cell proliferation, replacing damaged cells and restoring epithelial integrity, as previously reported in larvae exposed to sublethal *Bt* doses or in *Bt*‐resistant insect's strains.^43^, ^44^, ^45^

However, although this regenerative response limits mortality, the gut damages observed can still alter the gut environment and microbiota homeostasis, since *Bt*‐induced lesions are associated with shifts in microbial community composition.^5^, ^46^, ^47^ Given the tight interconnection between gut microbiota and host immune functions,^29^, ^48^, ^49^ we hypothesized that the limited gut damage observed could affect systemic immunity. Then, based on this hypothesis, we wanted to assess the occurrence of any impairment of cellular immunity in *Bt*‐larvae. Indeed, a pronounced reduction in phagocytic and nodulation activity was observed, whereas encapsulation remained unaffected. This pattern aligns with the specific role of encapsulation, which primarily targets large invaders such as multicellular fungal entomopathogens^50^ or parasitoid eggs.^51^, ^52^ By leaving this defense barrier intact, *Bt* enhances its own pathogenic potential while remaining protected from possible non‐bacterial competitors that could otherwise co‐infect its host.

The implications of these findings are particularly relevant for crop protection. Several studies have shown that immunodeficiency in insects can be exploited to increase the effectiveness of biological control agents.^53^ Targeted suppression of immune responses has indeed proven to be an effective strategy to enhance the efficacy of bioinsecticides.^5^, ^28^, ^30^, ^43^, ^54^, ^55^, ^56^, ^57^ In line with this rationale, the reduced cellular immune response we observed significantly enhanced the killing activity of *Bt* applications on *S. littoralis* larvae fed on *Bt*‐plants, even on later instars which are not sensitive to *Bt* toxins.

This study provides proof‐of‐concept evidence that *Bt* endophytic colonization in tomato plants, achieved using registered commercial formulation, can modulate insect physiology and immunity in ways that enhance susceptibility to *Bt*‐based bioinsecticides under controlled laboratory conditions. Starting from the soil, *Bt* can establish itself within the plant, forming a mutualistic interaction that benefits both organisms; by leveraging controlled artificial colonization, *Bt* can be directly integrated into plant tissues, thereby providing a complementary delivery route to conventional sprays. This intimate relationship may contribute to increase plant resilience to pest attacks by enhancing plant defence responses^58^ and ensuring active bacterial presence in plant tissues, potentially reducing the necessity to spray either the same bioinsecticide or even synthetic insecticides.

Most notably, leveraging a registered *Bt* formulation could accelerate translation of this endophytic delivery strategy, supported by prior regulatory and safety assessments, as the product has already undergone full regulatory approval and comprehensive safety evaluations for environmental and toxicological compliance. The combined action of endophytic *Bt* within the plant and subsequent low‐dose foliar applications of the same formulation provides a coordinated dual action against larval instars, with possible implications for resistance management that warrant dedicated investigation. In addition, this integrated approach may increase the susceptibility of later larval stages, which are often less responsive to spray‐based *Bt* treatments, thereby, potentially improving the overall insecticidal performance of *Bt‐*based treatments.

Altogether, the sequential use of *Bt* root drenching in young plants followed by foliar applications could represent a promising complementary strategy for managing *S. littoralis* populations. While this work supports the biological suitability and mechanistic basis of such an approach under controlled laboratory conditions, its practical implementation as a crop protection strategy requires further validation. Field‐based studies will be essential to assess the stability of endophytic colonization, its consistency across environmental contexts, and its effectiveness under agronomic conditions where multiple biotic and abiotic factors may influence plant‐microbe‐insect interactions.^59^

## CONFLICT OF INTEREST

The authors declare that they have no known competing financial interests or personal relationships that could have appeared to influence the work reported in this article.

## AUTHOR CONTRIBUTIONS

MGDL, GJ, ER, investigation, data analysis, writing. IDL conceptualization, investigation, data analysis and interpretation, supervision, writing – original draft, writing – review and editing. AB, investigation, writing. DB, investigation, writing. GT, conceptualization, supervision, writing – review and editing. MCD, conceptualization, supervision, writing – original draft. FP, conceptualization, supervision, writing – original draft, writing – review and editing, funding acquisition, project coordination. All authors critically reviewed the manuscript and gave final approval for submission.

## Supporting information


**Table S1.** Primer sequences and their characteristics used for diagnostic PCR in the detection of *Bt* in plant tissues (first line) and in *S. littoralis* gut (second and third lines).
**Fig. S1. Agarose gel electrophoresis 1% (w/v) of Cry1 and Vip3 genes amplification products obtained from RNA extraction**. PCR amplification of *Cry1* and *Vip3* genes from midgut of *Bt‐*larvae. Lane 1: *Cry* gene; Lane 2: *Vip* gene; Lane 3: molecular marker.

## Data Availability

The data that support the findings of this study are available from the corresponding author upon reasonable request.
