# Supplementary material for: Bacillus thuringiensis and its pest control potential as endophyte
Source: Pest Manag Sci. 2026 Mar 28;82(7):6931–9. doi: 10.1002/ps.70771 (PMC13240705; doi:10.1002/ps.70771)
Supplement: Supplementary file 1 — Table S1. Primer sequences and their characteristics used for diagnostic PCR in the detection of Bt in plant tissues (first line) and in S. littoralis gut (second and third lines). Fig. S1. Agarose gel electrophoresis 1% (w/v) of Cry1 and Vip3 genes amplification products obtained from RNA extraction. PCR amplification of Cry1 and Vip3 genes from midgut of Bt‐larvae. Lane 1: Cry gene; Lane 2: Vip gene; Lane 3: molecular marker. [file PS-82-6931-s001.docx]

**Supporting Methods 1. Isolation, molecular identification, and preparation of *Bacillus thuringiensis* inocula**

*Bacillus thuringiensis* subsp. *aizawai* strain A*BT*S-1857 (*Bt*) was isolated from the commercial product XenTari^®^ (Valent BioSciences Corporation, Libertyville, Illinois, USA), which contains several Cry toxins (Cry1Aa, Cry1Ab, Cry1Ca, Cry1 Da and Cry2Ab) and it’s active on *Spodoptera* spp.

Since *Bt* occurs either as vegetative cells or as spores, depending on growth conditions, distinct inocula were prepared to assess the most effective bacterial form for tomato plant colonization.

A starter culture was obtained by suspending 0.1 g of XenTari^®^ in 10 mL of sterile distilled water (ddH_2_O). Serial dilutions (from 10^-3^ to 10^-5^) were plated (100 µL) onto Luria Bertani (LB) agar (Lennox L Agar, Invitrogen™ - Thermo Fisher Scientific, Waltham, Massachusetts, USA) into Petri dishes and incubated at 30^o^C for 24 hours.

Distinct colonies were morphologically characterized following the criteria described by Rabinovitch et al.¹ To confirm their molecular identity, colonies displaying *Bt*-like morphology were individually collected using sterile inoculation loops, suspended in nuclease-free water, and subjected to PCR amplification of the *Cry1* gene using the primers reported in Table 1, which were also employed for the verification of endophytic colonization in tomato tissues. PCR reactions were performed using a commercial PCR kit (Thermo Fisher Scientific, Waltham, MA, USA).² PCR products were separated on 1.5% (w/v) agarose gels (Euroclone S.p.A., Milan, Italy) stained with GelRed (Thermo Scientific, Waltham, MA, USA) and visualized under UV illumination using a ChemiDoc™ Imaging System (Bio-Rad, Hercules, CA, USA) to confirm the presence of the expected amplicon band. The resulting amplicons were subsequently used as positive controls in all molecular assays.

A single checked colony was cultured in 5 mL of LB broth (Miller’s LB Broth Base, Invitrogen™, Lenexa, Kansas, USA) at 30 °C and 150 rpm for 24 hours on a benchtop orbital shaker (MaxQ™ 4450, Thermo Scientific™, Marietta, Ohio, USA).

Two inocula were prepared by transferring 1 mL of the starter culture into 100 mL of growth medium (25 g/L LB broth; 1 g/L KH_2_PO_4_; 1 mg/L CaCO_3_; 1 mg/L MgSO*7H_2_O; 0,1 mg/L FeSO_4_*7H_2_O; 21,5 mg/L MnSO_4_*H_2_O; 0,1 mg/L ZnSO_4_*7H_2_O),^3^ and incubated under the same conditions described above.

Samples were collected in 50 mL sterile conical tubes (Falcon™, Corning Inc., Corning, NY, USA) after 1 and 7 days, to obtain vegetative cells and spores, respectively. Such samples were centrifuged at 4,000 rpm for 30 minutes at 4^o^C (Multispeed Refrigerated Centrifuge PK 121 R, ALC, Cologno Monzese, Milan, Italy), then the pellets were resuspended in 0.1% Tween 20 (Promega Corporation, Madison, WI, USA) in ddH_2_O. The 7-day sample was further heat-treated at 70^o^C for 15 minutes to remove vegetative cells.

Bacterial concentrations were estimated by plating serial 10^-3^ - 10^-5^ dilutions (100 µL) in triplicate on LB agar plates, incubating at 30°C for 24 h, and counting colony-forming units (CFU/mL). To verify minimal negligible sporulation in the 1-day culture, 1 mL of this solution was heat-treated, serially diluted, and plated as above. Colonies from heat-resistant spores were counted after 24 hours of incubation, allowing for the calculation of the sporulation frequency as the ratio of heat-resistant spores to the total cell count from the untreated culture.

The following inoculum suspensions were prepared for plant treatments: (i) 1 × 10⁷ vegetative cells/mL; (ii) 1 × 10⁷ spores/mL; (iii) 1 × 10⁷ equimolar mixture of vegetative cells and spores/mL; (iv) 1 mg/mL XenTari®; all in 0.1% Tween 20 in ddH₂O. Control plants (C) received only 0.1% Tween 20 in ddH₂O.

**Supporting Methods 2. Tomato plant inoculation**

Tomato seeds (*Solanum lycopersicum* L., cultivar “Dwarf San Marzano”) were surface-sterilized in EtOH 70% for 3 minutes, rinsed, immersed in NaOCl 1% for 5 min, and washed three times with sterile ddH₂O. Seeds were germinated on sterile Whatman® filter paper (Sigma-Aldrich, Darmstadt, Germany) moistened with sterile ddH_2_O, placed in five separate Petri dishes, and maintained in a growth chamber in the dark at 25 ± 1 °C and 70 ± 5% relative humidity (RH). Soon after roots emergence, 1 mL of each inoculum was added to a separate Petri dish, so that each of the five dishes corresponded to a distinct treatment. The dishes were subsequently maintained in a growth chamber at 25 ± 1 °C, 70 ± 5% RH, and a 16:8 h light/dark photoperiod until seedling emergence. Two days later, the seedlings were individually transplanted into polystyrene trays (96 well) containing commercial potting soil (Universal Potting Soil, Floragard, Oldenburg, Germany) and watered with 2 mL of the corresponding suspension. The treatment was repeated one week later. Three-week-old plants were transferred to 10 cm pots, maintained under the same conditions, irrigated three times a week with tap water.

**Supporting Methods 3. Assessment of endophytic colonization**

To assess endophytic colonization*,* 5 tomato plants per treatment were randomly selected two weeks after the second inoculation (4-week-old plants), carefully washed under running tap water, primarily performed on roots to remove adhering coarse soil particles, and surface-sterilized (1% NaOCl for 5 min; 70% EtOH for 1 min; three rinses in sterile ddH₂O). Sterilization efficacy was checked by plating 100 µL each of the final rinse water on LB agar plates, in triplicate. Sterilized tissues were sectioned, plated on LB agar, and incubated at 30°C in the dark for 48 h, to monitor bacterial growth. Colonization was expressed as (i) the percentage of colonized plants over the total number treated, and (ii) the colonization frequency, calculated as the percentage of colonized tissue sections per plant relative to the total number of tissue sections,^3^ according to the following formula: Frequency of colonization (%) = [(number of colonized tissue sections)/(total number of tissue sections)] × 100.^4^

**Supporting Methods 4. Morphological and molecular verification of endophytic colonization in tomato plant**

Bacterial colonies emerging from surface-sterilized tissues were sub-cultured on LB agar and incubated at 30 °C for 24 h to obtain pure cultures. Distinct colonies were morphologically characterized as previously described, ¹ and their molecular identity was verified by PCR amplification (see Supporting Methods1) of the *Cry1* gene using primers available in literature^3^ , which are reported in Supporting Table 1. PCR products were separated on 1.5% (w/v) agarose gels and visualized under UV illumination using a ChemiDoc™ Imaging System (Bio-Rad, Hercules, CA, USA) to confirm the presence of the expected amplicon band.

As additional evidence of successful endophytic colonization, *Cry1* gene amplification was carried out directly on leaf tissues from plants used for subsequent insect bioassays. The fifth fully expanded leaf (absent at the time of inoculation and therefore not directly exposed to the initial treatment) was collected from six-week-old plants prior to the bioassays, to verify systemic colonization and ensure that only *Bt*-colonised plants were used in the experiments. Leaf samples were immediately frozen in liquid nitrogen and stored at -80 °C until DNA extraction, performed according to a previously established protocol. ⁵ DNA concentration and purity were assessed spectrophotometrically at 260 nm and 260/280 nm (Varioskan Flash, Thermo Fisher Scientific, Vantaa, Finland). Extracted DNA was stored at −20 °C until PCR analysis, conducted following the same procedure used for *Bt* identification (see Supporting Methods 1) and employing the primers listed in Supporting Table 1. The amplification yielded the expected *Cry1*-specific fragment, confirming the presence of *B. thuringiensis* within plant tissues. Only plants testing positive for *Bt* colonization were subsequently used in the feeding bioassays.

**Supporting Methods 5. Insect bioassays on leaves of tomato plants colonized by *Bt***

*Spodoptera littoralis* experimental larvae, derived from a colony established at the University of Napoli Federico II (Department of Agricultural Sciences, Laboratory of Entomology "Ermenegildo Tremblay", Portici-NA, IT). The colony is refreshed annually with field-collected individuals to preserve genetic variation. Larvae were reared under controlled environmental conditions (25 ± 1 °C, 70 ± 5% RH, and a 16:8 h light/dark photoperiod) as previously described.^6^

Groups of newly hatched *S. littoralis* larvae were reared in plastic boxes (30 ×40 ×15 cm), bottom lined with 50 mL of 1.5% (w/v) agar and fed with sub-apical leaves excised from 4 weeks-old *Bt-*plants (*Bt-*larvae) or of C-plants (C-larvae). Leaves were randomly selected from the second whorl and carefully detached from plants that had been left undisturbed for at least three days, to avoid any confounding induction of defense responses caused by mechanical damage.^7,8^

*S. littoralis* larvae were separated into six groups of 20 larvae each per treatment (*Bt-*larvae, C-larvae), and transferred into plastic multi-well rearing trays (RT32W, Frontier Agricultural Sciences, Pitman, NJ, USA), bottom-lined with 1 mL of 1.5% (w/v) agar and closed with perforated plastic lids (RTCV4, Frontier Agricultural Sciences, Pitman, NJ, USA). The larvae were fed daily with sub-apical leaves of 6-week-old experimental plants. The bioassay was carried out in an environmental chamber, at 25 ± 1 °C, 70 ± 5% RH, and photoperiod of 16:8 h light/dark. Larval survival and development were daily monitored.

From each experimental group, 16 synchronized, newly molted 3^rd^ instar larvae were collected immediately after moulting from surviving second instars, individually weighed, and transferred into separate wells of plastic trays as described above. Each larva was provided daily with a 4 cm² leaf disk, replaced every 24 h.

To assess adult fertility (number of eggs laid per female) and egg viability (hatching rate), each emerged female was paired with two males for 24 h and provided with a 50% (v/v) honey–water solution. After mating, females were individually isolated in plastic boxes (40 × 30 × 20 cm) and supplied with the same diet throughout the oviposition period.

**Supporting Methods 6. RNA extraction, cDNA synthesis, and PCR analysis to assess *Bt* presence in larva midgut**

Second-day 6^th^ instar *Bt*- and C-larvae were cold-anesthetized and surface-sterilized by immersion in 4% NaOCl for 3 min, followed by 70% (v/v) EtOH in ddH₂O for 3 min, and finally rinsed with sterile ddH₂O. Larvae were blotted on sterile filter paper and dissected in a Petri dish under a horizontal-flow hood to isolate the midgut. Each larva was opened longitudinally to remove fat body and tracheae, and the midgut was carefully excised with fine microscissors (Fine Science Tools GmbH, Germany). Tissues were washed in sterile 1× PBS (137 mM NaCl, 2.7 mM KCl, 10 mM phosphate buffer, pH 7.4), immediately transferred into TRIzol® (Thermo Fisher Scientific, Grand Island, NY, USA), and stored at –80 °C until RNA extraction.

Total RNA was extracted from larval tissues in TRIzol^®^ following the manufacturer's protocol, and treated with Turbo DNase (Invitrogen, Carlsbad, California, USA) to remove residual genomic DNA. RNA integrity and concentration were evaluated spectrophotometrically as previously described for DNA samples. Complementary DNA (cDNA) was synthesized from 1 µg of total RNA using the High-Capacity cDNA Reverse Transcription Kit (Thermo Fisher Scientific, Carlsbad, CA, USA).

To verify the presence of *Bt* subsp. *aizawai*, the strain used to colonize tomato plants, as indirect evidence of the bacterium’s persistence and activity in the larval midgut, two genes were selected: *Cry1* and *Vip3*. The gene sequences were retrieved from the GenBank database (National Center for Biotechnology Information - NCBI, Bethesda, MD, USA) (**Supporting Table 1**). For both genes multiple sequences were aligned using Clustal Omega (version 1.2.2; clustal.org, Conway Institute, University College Dublin, Ireland) to identify conserved regions suitable for primer design. Specific oligonucleotides were then designed with Primer3 software (v. 0.4.0; Cambridge, MA, USA), using the following parameters: primer length 18–24 nucleotides, melting temperature (Tm) 54–60 °C, and GC content 40–60%. Primer specificity was verified with BLAST^®^ 2.9.0 (NCBI, Bethesda, MD, USA) to rule out any significant similarity with non-target sequences. The designed primer pairs (listed in Supporting Table 1) amplify fragments of 290 bp for *Cry1* and 334 bp for *Vip3* gene transcript.

PCR amplification of the synthesized cDNA was carried out using 0.1 µM of each specific primer and the previously described RT-PCR kit. A 100 bp DNA ladder was used as a molecular weight marker, and amplification products were visualized under UV light as previously described. RT-PCR products were further verified by sequencing to confirm their identity.

| **Diagnostic**  **tool** | **Gene** | **Name** | **Sequence (5’-3’)** | **Melt. temp.**  **(°C)** | **Amplicon length (bp)** | **Accession** |
| --- | --- | --- | --- | --- | --- | --- |
|  | *Cry1*  *(plant)* | Cry1-FW  Cry1-RW | GCGTAGAAGAGGAAGTCTATCCAA  ATATCCTCGATTACGAGAAGTGTACG | 54 | 105 | M73254 |
|  | *Cry1* | Cry1FW  Cry1RW | TGTAGAAGAGGAAGTCTATCCA  TATCGTTTTCTGGGAAGTA | 52 | 290 | X06711 |
|  | *Vip3* | VIP3-FW  VIP3-RV | TGGATGGGGTGAATGGAAGC ATCCTTTGATACGCAGGTGT | 54 | 334 | KY883694.1 |

**Supplementary Supporting Table 1. Primer sequences and their characteristics used for diagnostic PCR in the detection of *Bt* in plant tissues (first line) and in *S. littoralis* gut (second and third lines)**

**Supporting Methods 7 for midgut morphological analysis**

*Bt-*larvae and C-larvae at 1^st^, 2^nd^, and 3^rd^ day of the second instar were ice-anesthetized and dissected by removing the head and the caudal region. Samples were fixed in 4% (v/v) glutaraldehyde in 0.1 M sodium cacodylate buffer, pH 7.4, for 2 h at room temperature (RT) and stored overnight at 4°C before embedding in Epon resin.^9^ Semi-thin sections (600 nm-thick) were stained with crystal violet and basic fuchsin and observed with an Eclipse Ni-U microscope (Nikon Instruments Inc., Melville, New York, USA), equipped with a DS-5 M-L1 digital camera system (Nikon). Ultra-thin sections (70 nm-thick) were stained with lead citrate and uranyl acetate and observed with a JEM-1400 transmission electron microscope (Jeol Ltd., Akishima, Tokyo, Japan) equipped with Morada digital camera (Olympus, Hachioji, Tokyo, Japan) - CRIETT, University of Insubria. Five samples per experimental group were examined.

**Supporting Methods 8. Haemocyte viability, encapsulation, nodulation and phagocytosis assays**

All assays were performed on 6^th^ instar larvae, which were ice-anesthetized and surface-sterilized with 70% ethanol before their processing.

*In vivo* encapsulation assays were performed using 60 CM Sepharose Fast Flow chromatography beads (Pharmacia, Uppsala, Sweden), suspended in 1 × PBS. Beads were injected into the larval haemocoel through the neck membrane using a precision Hamilton Microliter syringe (Reno, Nevada, USA, 702 RNR 25 μL, gauge 22s, length 55 mm, needle 3). After 24 h, larvae were dissected to recover the beads, which were observed under a stereomicroscope (SteREO Discovery.V8,

Zeiss, Jena, Germany). The encapsulation index was calculated using an index that accounts for both the degree of encapsulation on each bead and the relative abundance of beads with different encapsulation levels^11^.

For the *in vivo* nodulation assays, experimental larvae received an injection into the haemocoel, through the neck membrane, of 2 × 10⁶ *Escherichia coli* cells suspended in 5 μL of 1× PBS, using a Hamilton 1701 RN syringe (10 μL, 26s gauge, 55 mm, point style 3). After 18 h, a thoracic leg was severed and the exuding haemolymph was collected, then immediately diluted 1:1 with ice-cold Mead anticoagulant buffer (98 mM NaOH, 145 mM NaCl, 17 mM EDTA, and 41 mM citric acid, pH 4.5). Haemocyte nodules present in the haemolymph were counted under a microscope (Axioskop; Carl Zeiss Microscopy, Jena, Germany) using a Bürker chamber.

To evaluate haemocyte phagocytic activity *in vitro*, haemolymph was collected as described above and transferred into 1.5 mL Eppendorf tubes containing ice-cold PBS (2:1, v/v). A PBS suspension of 2 × 10⁷ fluorescein-conjugated *E. coli* cells (K-12 strain BioParticles; Life Technologies, Eugene, OR, USA) was prepared according to the manufacturer’s instructions, added to the haemolymph samples (2:1, v/v), and incubated at room temperature for 10 min. Samples were then loaded into a Bürker chamber, and both total and fluorescent haemocytes were counted under a fluorescence microscope (Axioskop 20; Carl Zeiss Microscopy, Jena, Germany).

For both the nodulation and phagocytosis assays, total and dead haemocytes were quantified using a trypan blue exclusion test. An aliquot of haemolymph from experimental larvae was mixed 2:1 (v/v) with a 0.4% (w/v) trypan blue solution in water (Sigma-Aldrich, St. Louis, MO, USA) and counted in a Bürker chamber. The total haemocyte number in *Bt*- and C-larvae was determined following the manufacturer’s instructions. Samples with viability below 98% were discarded; fewer than 1% of samples showed viability below this threshold.

**Supporting Results 1. Assessment of *Bt* endophytic colonization through RT-PCR**

RT-PCR analysis confirmed the active presence of *Bt* in the midgut of experimental larvae feeding on *Bt-*plants, revealing the expression of insecticidal *Cry* and *Vip* genes, even in surviving 6^th^ instar larvae (**Supporting Figure 1**).


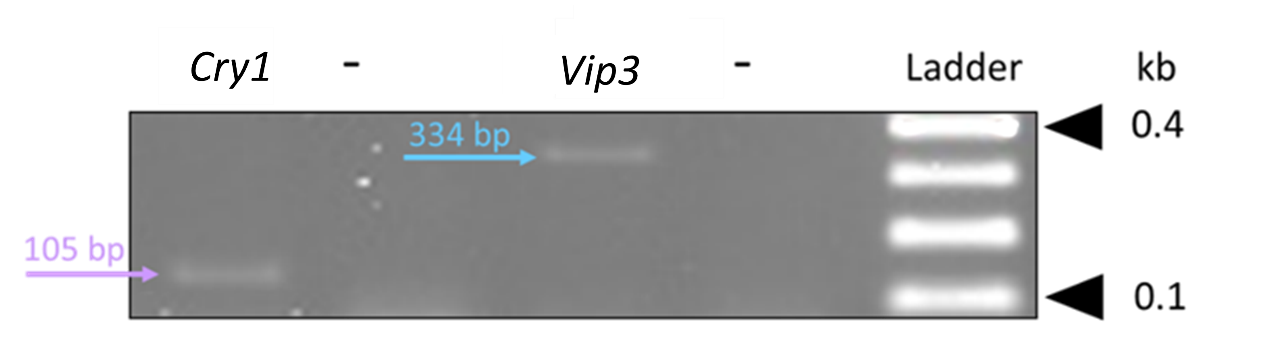


**Figure 1. Agarose gel electrophoresis 1% (w/v) of *Cry1* and *Vip3* genes amplification products obtained from RNA extraction.** PCR amplification of *Cry1* and *Vip3* genes from midgut of *Bt-*larvae. Lane 1: *Cry* gene; Lane 2: *Vip* gene; Lane 3: molecular marker.

**Supporting Bibliography**

1 Rabinovitch L, Vivoni AM, Machado V, Knaak N, Berlitz DL, Polanczyk RA and Fiuza LM, *Bacillus thuringiensis* characterization: morphology, physiology, biochemistry, pathotype, cellular, and molecular aspects. In: Fiuza LM, Polanczyk RA and Crickmore N (eds), *Bacillus thuringiensis and Lysinibacillus sphaericus characterization and use in the field of biocontrol*. 1st ed. Cham: Springer, pp. 1–18 (2017). doi:10.1007/978-3-319-56678-8_1.

2 Cerón J, Ortíz A, Quintero R, Güereca L and Bravo A, Specific PCR primers directed to identify cryI and cryIII genes within a Bacillus thuringiensis strain collection. Appl Environ Microbiol 61(11):3826–3831 (1995). doi:10.1128/aem.61.11.3826-3831.1995.

3 Monnerat RG, Soares CM, Capdeville G, Jones G, Martins ÉS, Praça L, Arrivabene B, Braz SV, dos Santos R and Berry C, Translocation and insecticidal activity of *Bacillus thuringiensis* living inside of plants. *Microb Biotechnol* **2**(4):512–520 (2009). doi:10.1111/j.1751-7915.2009.00116.x.

4 Petrini O, Fungal endophytes of tree leaves. In: Andrews JH and Hirano SS (eds), Microbial Ecology of Leaves. Brock/Springer Series in Contemporary Bioscience. Springer, pp. 179–197 (1991). doi:10.1007/978-1-4612-3168-4_9.

5 Cubero OF, Crespo A, Fatehi J and Bridge PD, DNA extraction and PCR amplification method suitable for fresh, herbarium‑stored, lichenized, and other fungi. *Plant Syst Evol* **216**(3–4):243–249 (1999). doi: 10.1007/BF01084401.

6 Di Lelio I, Varricchio P, Di Prisco G, Marinelli A, Lasco V, Caccia S, Casartelli M, Giordana B, Rao R, Gigliotti S and Pennacchio F, Functional analysis of an immune gene of *Spodoptera littoralis* by RNAi. *J Insect Physiol* **64**(1):90–97 (2014). doi: 10.1016/j.jinsphys.2014.03.008.

7 Glauser G, Grata E, Dubugnon L, Rudaz S, Farmer EE and Wolfender JL, Spatial and temporal dynamics of jasmonate synthesis and accumulation in *Arabidopsis* in response to wounding. *J Biol Chem* **283**(24):16400–16407 (2008). doi:10.1074/JBC.M801760200.

8 Yan L, Zhai Q, Wei J, Li S, Wang B, Huang T, Du M, Sun J, Kang L, Chang‑Bao Li and Chuanyou Li, Role of *Tomato lipoxygenase D* in wound-induced jasmonate biosynthesis and plant immunity to insect herbivores. *PLoS Genet* **9**(12):e1003964 (2013). doi:10.1371/journal.pgen.1003964.

9 Franzetti E, Romanelli D, Caccia S, Cappellozza S, Congiu T, Rajagopalan M, Grimaldi A, de Eguileor M, Casartelli M and Tettamanti G, The midgut of the silkmoth *Bombyx mori* is able to recycle molecules derived from degeneration of the larval midgut epithelium. *Cell Tissue Res* **361**(2):509–528 (2015). doi:10.1007/s00441-014-2081-8.

10 Wang Z, Yang Y, Li S, Ma W, Wang K, Soberón M, Yan S, Shen J, Francis F, Bravo A and Zhang J, JAK/STAT signaling regulated intestinal regeneration defends insect pests against pore‑forming toxins produced by *Bacillus thuringiensis*. *PLoS Pathog* **20**(1):e1011823 (2024). https://doi.org/10.1371/journal.ppat.1011823.

11 Li XY, Jin LJ, Lu YN, Zhen YH, Li SY, Wang LH and Xu YP, Chitosan–alginate microcapsules for oral delivery of egg yolk immunoglobulin (IgY): effects of chitosan concentration. *Appl Biochem Biotechnol* **157**:778–787 (2009). https://doi.org/10.1021/jf062900q.
